# Ischemic preconditioning: exploring local ergogenic mechanisms in non-fatiguing voluntary contractions

**DOI:** 10.3389/fphys.2025.1542394

**Published:** 2025-03-25

**Authors:** Ruben Allois, Raffaele Pertusio, Pasquale Pagliaro, Silvestro Roatta

**Affiliations:** ^1^ Department of Neuroscience, University of Torino, Torino, Italy; ^2^ Department of Clinical and Biological Sciences, University of Torino, Torino, Italy

**Keywords:** ischemic pre-conditioning, tissue oxygenation, blood flow, no-fatigue, performance

## Abstract

**Background:**

IPC has been suggested to boost skeletal muscle performance, though its effectiveness remains controversial. This study evaluates whether IPC influences local hemodynamic responses and surface electromyographic (sEMG) activity during non-fatiguing voluntary sustained and intermittent contractions.

**Methods:**

Ten male participants were subjected to IPC (3 cycles, 5-min ON/5-min OFF right arm ischemia, cuff pressure: 250 mmHg) and SHAM (same protocol at 20 mmHg) in two different sessions. Near-infrared spectroscopy was used to monitor tissue oxygenation (TOI) and deoxy-hemoglobin (HHb) in extensor and flexor forearm muscles. sEMG was also recorded. Measurements were taken during sustained (20-s duration) and intermittent (5 s ON/5 s OFF) isometric contractions at 20, 30, and 40% of the maximal voluntary contraction. These non-fatiguing exercise tasks were performed before and 30 min after the IPC/SHAM intervention.

**Results:**

sEMG exhibited a significant increase post vs. pre-treatment in both IPC and SHAM in extensors. A significant decrease in TOI at rest was noted pre vs. post-treatment for both IPC and SHAM (p < 0.01). In general, no main effect of treatment was observed, except for HHb changes during contraction in extensor muscles, associated with no effect of time and no time-treatment interaction. All variables exhibited a main effect of force level (p < 0.05), with no interaction with treatment or time.

**Conclusion:**

IPC had no effect on hemodynamic and electromyographic variables during sustained and intermittent handgrip. These results do not support IPC-related ergogenic effects at the muscle level, aligning with previous findings on electrically stimulated contractions.

## 1 Introduction

Ischemic preconditioning (IPC) is a minimally-invasive technique that involves short episodes of ischemia followed by reperfusion (Caru et al., 2019). This methodology has been employed to safeguard tissues and organs from the possible damage caused by prolonged ischemia and reperfusion injury (Eltzschig and Eckle, 2011). Besides this protective effect, IPC has also been claimed to improve muscle function and sports performance.

In this field, standard IPC protocols consist of a 3 or 4-set of 5-min unilateral or bilateral limb ischemia (alternately performed on the two sides), obtained by proximal cuff inflation at supra-systolic pressure (Allois et al., 2023; Landman et al., 2022; Paradis-Deschênes et al., 2017; Valenzuela et al., 2021). While it is accepted that IPC may improve performance in endurance exercise (Salvador et al., 2016) and increase time to exhaustion/failure (Allois et al., 2023), the improvement in muscle force during non-fatiguing conditions is debated. For instance, IPC increased one repetition maximum (1-RM) force by ∼2.4 kg in bench press exercise compared to baseline, while the 1.8 kg increase in the SHAM group was not significant, although the IPC-SHAM difference was not explicitly tested (Rodrigues et al., 2023). Along the same line, in exercises like the bench press and leg extension, maximum strength was reported to increase in both IPC (220 mmHg) and SHAM (20 mmHg) groups, compared to the control group (no treatment), with no significant difference between the first two groups (Telles L. G. et al., 2022). Conversely, many studies failed to evidence a significant IPC-induced increase in force in non-fatigued muscles, even during the same bench-press exercise (Valenzuela et al., 2021).

Note that, although the 1-RM bench press requires a maximal effort and is, *per se*, a fatiguing task, it is taken on by non-fatigued muscles and, as a one-shot exercise, it does not require the subject to cope with a growing fatigue condition as with endurance exercise. On the other hand, it engages virtually the whole body, making it difficult to detect the origin of the possible ergogenic effect. Adopting exercise models involving single or small muscle groups may be a better strategy to investigate local mechanisms while limiting systemic effects. One example is the isometric handgrip, often adopted, also in IPC investigations. Some studies have suggested that IPC may improve acute handgrip strength in both elderly and active individuals, showing potential benefits in muscle performance (Valenzuela et al., 2021). This effect would be linked to IPC’s ability to increase blood flow and muscle oxygenation during and after ischemia, thereby enhancing muscle contractility (Telles L. G.daS. et al., 2022). However, not all studies agree on this point: another study found no significant IPC impact on handgrip strength exercise compared to placebo (Rodrigues et al., 2023), highlighting the need for additional investigations.

A non-adequately controlled placebo effect has been suggested to account to for the variability in results (Souza et al., 2021). Intending to exclude the influence of placebo effects we previously investigated the influence of IPC on electrically stimulated contractions (Allois et al., 2024). The stimulation protocol included single pulses, short trains of stimuli at different frequencies, and short bursts. However, none of these patterns resulted in an augmentation of the developed force after IPC. On the contrary, a moderate but significant force decrease occurred after both IPC and SHAM treatments, suggesting that ergogenic effects observed in other experimental conditions could be ascribed to placebo effects. It may be objected that the electrically-stimulated contraction is not a physiological muscle contraction, as it does not reproduce the orderly recruitment of motor units. We considered that one way of investigating voluntary contractions while excluding the placebo effect was by engaging the subjects in constant-force submaximal, rather than maximal contractions. We hypothesized that, in this case, IPC-induced changes in muscle contractility or metabolism would be revealed by changes in electromyographic (EMG) activity or tissue oxygenation.

Thus, in the present study, we aimed to reassess the effects of ischemic preconditioning on controlled submaximal isometric contractions. By maintaining a constant and low (i.e., easily sustainable) force level for a brief period of time we also prevented the onset of fatigue. In addition, to discriminate whether possible ergogenic effects are due to changes in the contractility/metabolism of the muscle cells or the blood supply capacity, both continuous (20 s) and intermittent (5s ON and 5s OFF) contractions were investigated, given that only the latter would benefit of possibly improved blood supply during the hyperemic phase in-between contractions, while isometric contractions basically occur in ischemic conditions.

## 2 Materials and methods

### 2.1 Subjects and study design

Ten male subjects were enrolled (age: 27 ± 10 years, mass: 71.2 ± 10.0 kg, height: 177.7 ± 6.7 cm; BMI: 22.5 ± 2.8 kg/m^2^. Participants were eligible for the study if they met the following criteria: healthy individuals with no prior history of medical conditions, non-smokers, and free of any medications that might affect the outcome measures. Participants were excluded if they had any neuromuscular impairment that would prevent them from completing the study procedures. The study was conducted in agreement with the principles of the Declaration of Helsinki and under the approval of the Ethics Committee of the University of Torino (Prot. 125,507, 16 February 2024). The subjects gave written informed consent.

The sample size was calculated based on our previous study. An effect size of 0.5 was estimated for EMG in constant-force submaximal contractions, considering that a 15% decrease in force was observed post-IPC (at 12-Hz stimulation) (Allois et al., 2023) and assuming a linear EMG-force relation. In addition, we hypothesized a standard deviation of about 30% of the mean for the voluntary sub-maximal contractions of the present study and a power of 0.8 (calculated with: G-Power ver. 3.1.9.6 (Heinrich-Heine-Universitat Düsseldorf, Düsseldorf, Germany)

### 2.2 Experimental set-up

**FIGURE 1:**
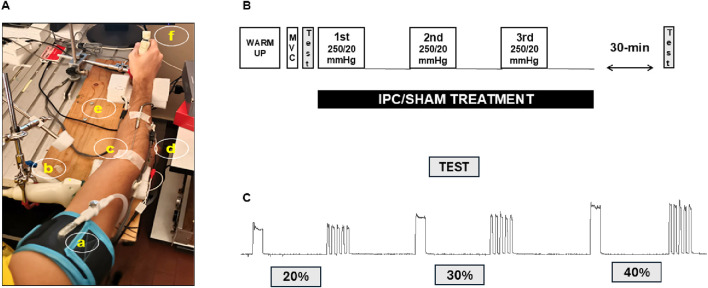
**(A)** Experimental Setup. The subject sits, leaning a bit forward, the elbow flexed at about 120°. Pressure cuff (a); ultrasound probe (b); temperature probe over the brachial muscle (c) near-infrared spectroscopy and the sEMG electrodes over extensor (d) and flexor muscles (e); handgrip (f). **(B)** Experimental Protocol. Includes warm-up, maximal voluntary contraction (MVC) assessment, pretreatment exercise test, 3-cycle treatment (IPC/SHAM), post-treatment exercise test**. (C)** Exercise test detail. Sustained and intermittent isometric contractions at 20, 30, and 40 %MVC, the order was randomized.

As can be observed in Figure 1A, an arm rest was designed for implementing isometric and intermittent voluntary contractions of the handgrip of the right hand. The subject sat, with back and arms rest (at the elbow and the wrist), elbows flexed at about 120° and the shoulder adducted and neutrally rotated. The handgrip is upheld by a flexible and steady mechanical arm to exclude a continuous load on forearm muscles.

The IPC/SHAM treatment was delivered to the right arm using a pneumatic cuff (The Occlusion Cuff® 8 × 75 cm, The Occlusion Cuff LTD, Somerset, United Kingdom) rapidly inflated to 250/20 mmHg by a custom system based on PC-driven proportional valve (ITV1010, produced by RTI s.r.l., located in Torino, Italy) supplied with compressed air (at 200 kPa), inflation time: about 2–3 s, deflation time: 3–5 s (Ferraresi et al., 2019; Messere et al., 2018).

During IPC, the complete occlusion of the brachial artery was continuously verified by absence of flow, measured by duplex ultrasound (MyLab 15, Esaote S.p.A., Genoa, Italy) with a linear probe (LA 523, Esaote, Genoa, Italy) positioned transversely at the upper arm, just distally to the cuff, with an insonation angle of about 60 deg.

The cutaneous temperature was continuously monitored by a metal probe placed over the brachioradialis muscle (TERZMI-I, Terzano and C s.r.l., Milano, Italy).

Arterial blood pressure (ABP) was continuously and non-invasively monitored by finger photoplethysmography (CNSystems, Medizintechnik GmbH, Graz, Austria).

The non-invasive monitoring of muscle oxygenation was performed using Near-Infrared Spectroscopy (NIRS) (NIRO-200X, Hamamatsu Photonics K.K, Shizuoka, Japan). Two NIRS probes, with an inter-optode distance of 3 cm, were positioned over the flexor and extensor forearm muscle (see Figure 1A). The device measures concentration changes in deoxygenated (HHb) and oxygenated (O2Hb) hemoglobin + myoglobin, expressed in µM/L*cm, using the standard Beer-Lambert methodology. Additionally, the NIRS device calculates the tissue oxygenation index (TOI), which represents the oxygen-saturated (hemoglobin + myoglobin) levels in percentage. This latter parameter is based on the spatially resolved methodology, which focuses the measurement in depth (muscle tissue) and is thus little affected by hemodynamic changes occurring in the superficial cutaneous tissue layer (Messere and Roatta, 2015; 2013a).

A custom-made device based on film sensors (FlexiForce A201 Tekscan, Boston, MA, United States of America) was used to measure handgrip force (Pertusio and Roatta, 2023). A visual feedback of the exerted force was provided to the subject to reach and maintain the requested force level, according to the experimental protocol.

Electromyographic (EMG) activity of the right forearm muscles was bipolarly recorded (QUATTRO, OT Bioelettronica Srl, Turin, Italy) with electrodes (F9089P/100, FIAB, Florence, Italy) placed transversally over flexor and extensor muscles, close and parallel to the NIRS probe. A reference electrode was placed over the right epicondyle. The skin was previously cleaned using an abrasive prepping gel.

### 2.3 Experimental protocol

The subjects participated in two different session in which they were subjected to one of two different treatments: the IPC treatment (3 × 5/5 min ischemia/reperfusion; 250 mmHg) or the SHAM treatment (3 × 5/5 min pseudo ischemia/reperfusion; 20 mmHg) in randomized order and separated by a minimum of 5 days. A scheme of the experimental protocol is given in Figure 1B, including warm-up, assessment of the maximum voluntary contraction (MVC), exercise test (pre-treatment), IPC/SHAM treatment, and exercise test (post-treatment). The subject was familiarized with the setup and invited to concentrate during the protocol. The warm-up included 3 exercises: 1) handgrip (3 sets of 10 repetitions); 2) sustained hand grip (3 sets of 5 repetitions, each lasting 20 s); 3) Intermittent hand grip (3 sets of 5 repetitions, with 5 s ON/5 s OFF). The rest period between sets was 1 min. Three separate maximal contractions lasting about 3 s and separated by 3 min of recovery were performed. The MVC value was determined as the highest force reached in these three attempts after low-pass filtering the signal with a 1-s width moving average and used to set the force reference for the exercise task as a percentage of the MVC. Five minutes after the last contraction, the exercise test was conducted, consisting of 3 sustained isometric contractions (lasting 20 s) and 3 intermittent contractions (5 s ON/5 s OFF, total duration 50 s) at 20%, 30%, and 40% of MVC. The 6 contractions were separated by a 3-min rest and their sequence was randomized. This protocol was defined based on preliminary experiments, as a trade-off of two requirements: 1) implementing a non-fatiguing task that could thus be performed both before and after the treatment, 2) achieving an exercise intensity sufficient to produce consistent changes in tissue oxygenation, in order to possibly detect IPC-induced changes also in this variable. Subjective fatigue perception (VAS) was recorded immediately after each sustained or intermittent contraction: the subject was asked to write a mark on a 10-cm bar (0 = no fatigue; 10 = maximum imaginable fatigue, complete exhaustion). The exercise test was repeated 30 min after the treatment (Caru et al., 2019; Salvador et al., 2016).

## 3 Data analysis

The average values of arterial blood pressure (ABP), temperature (TEMP) and tissue oxygenation index baseline in flexor (TOI_B-FLEX_) and extensor muscles (TOI_B-EXT_) were measured over a 20-s interval preceding the exercise test, before and after the IPC/SHAM treatment. Force and sEMG (pre-processed with notch-filter (50 Hz), rectification, DC removal and low-pass filtering with time constant: 0.1 s) levels were collected as time averages over the following intervals: from 1 to 19 s from the beginning of sustained contractions and from 0.5 to 4.5 s from the beginning of each bout in intermittent contractions, thus excluding from the analysis the transients associated with beginning and termination of the contraction (Allois et al., 2023). Changes in NIRS variables were differently calculated for sustained and intermittent contractions and for TOI and HHb: due to the fact that HHb, like O2Hb and tHb, presents a prominent movement artifact at the beginning and termination of each contraction (Felici et al., 2009), HHb changes had to be assessed either within or outside the contraction interval.

In sustained contractions changes were calculated as the difference between the last second of contraction and the baseline. This latter was calculated over 1-s intervals taken 10 s preceding the contraction (TOI) or 1 s after the beginning of the contraction (HHb). The TOI slope (TOI_S-FLEX;_ TOI_S-EXT_) was calculated by linear fitting over the interval [10–15 s] after the beginning of the contraction, where a most linear trend was expected (Felici et al., 2009).

In intermittent contractions, TOI change was assessed as the average calculated over the last contraction cycle (5 s contraction +5 s rest), compared to pre-exercise baseline and HHb change was calculated as the value post-contraction [1–2 s] after the end of the last contraction) minus the baseline (before the beginning of the contraction). TOI slope was assessed by linear interpolation of the tracing taken [1–4 s] after the beginning of each contraction bout, for the last 3 bouts and averaged.

All signal processing was performed with the acquisition and analysis software Spike2 and the measured values were collected in Excel sheets.

## 4 Statistical analysis

Statistical analysis was performed using SPSS 27.0 (SPSS Inc., Chicago, Illinois, United States). The results are presented as mean ± standard deviation in the text. The p-values smaller than 0.05 were considered significant. The normality of data was checked by Shapiro-Wilk’s test, while homoscedasticity was tested by Levene’s test. Subjects’ MVC was compared between treatments (IPC vs. SHAM) using an paired t-test. A two-way ANOVA for repeated measurements was performed to investigate the effect of treatment (IPC/SHAM) and time (PRE/POST treatment) on ABP, TEMP, and TOI in basal conditions (average over a 20-s interval positioned before the beginning of the sequence of exercise tasks). While a three-way ANOVA for repeated measurements was performed to assess the effect of treatment, time and force level (20%, 30% and 40% MVC) for variables collected during the exercise test: FORCE%, VAS, EMGs, TOI and HHb). The *post hoc* test with Bonferroni correction was performed to analyse pairwise differences.

## 5 Results

### 5.1 Basal conditions

The MVC, measured at the beginning of each session (IPC and SHAM), did not differ between the two sessions (IPC: 30.8 ± 10.5 kg vs. SHAM: 29.4 ± 9.9 kg).

No significant changes were observed in other variables measured PRE and POST treatment in each, session before the exercise, such as ABP (IPC: PRE 88.0 ± 6.1 and POST 88.7 ± 8.6 mmHg; SHAM PRE 83.4 ± 9.9 and POST 87.1 ± 6.5 mmHg) and TEMP (IPC: PRE 31.9 ± 0.9 and POST 31.2°C ± 1.0 C vs. SHAM PRE 32.3 ± 2.6 and POST 31.2°C ± 1.1 C).

The only significant effect of time (POST vs. PRE, in basal conditions), was exhibited by tissue oxygenation in both flexors (p < 0.01) and extensors (p = 0.051), with no effect of treatment session and no time-session interaction. On average, the TOI decreased by 1.6% ± 4.1% in flexors and 0.7% ± 5.6% in extensors.

### 5.2 Sustained contractions

**FIGURE 2:**
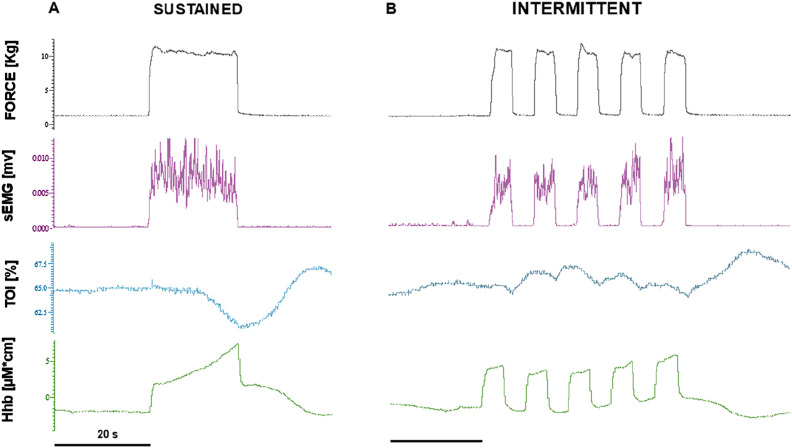
Original recordings from a representative subject, during sustained **(A)** and intermittent **(B)** contractions at 40% of MVC. From top to bottom: exerted force (FORCE), rectified surface electromyographic signal (sEMG), tissue oxygenation index (TOI), deoxy-hemoglobin (HHb), Note the movement artifacts in the HHb signal.

**FIGURE 3:**
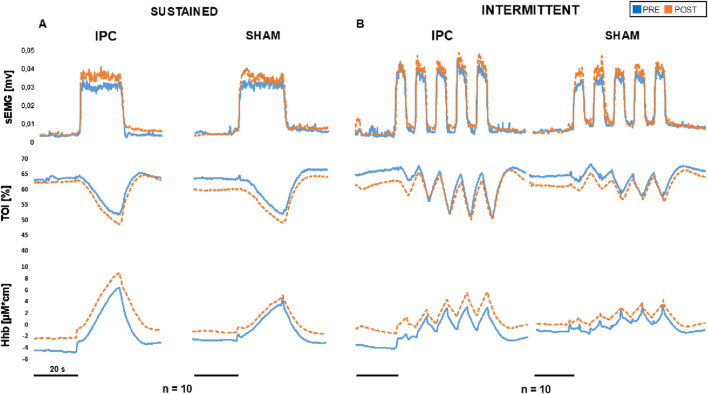
Rectified sEMG, TOI, and HHb signals in the extensor muscles during sustained **(A)** and intermittent contractions **(B)** at 40% MVC, before (blue) and 30 min after (red) the IPC/SHAM treatment. Each tracing represents the response averaged over all subjects (n = 10).

**FIGURE 4:**
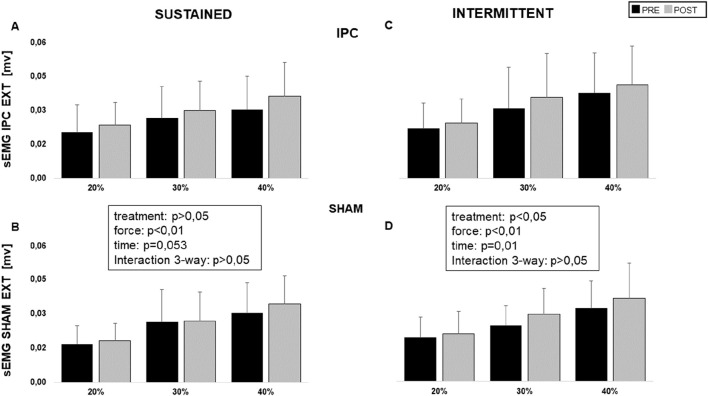
Average sEMG intensity reached by wrist extensor muscles in sustained and intermittent contractions at the different levels (20, 30, and 40% MVC) for the IPC **(A, B)** and SHAM groups **(C, D)**, before (black) and after (grey) treatment.

**FIGURE 5:**
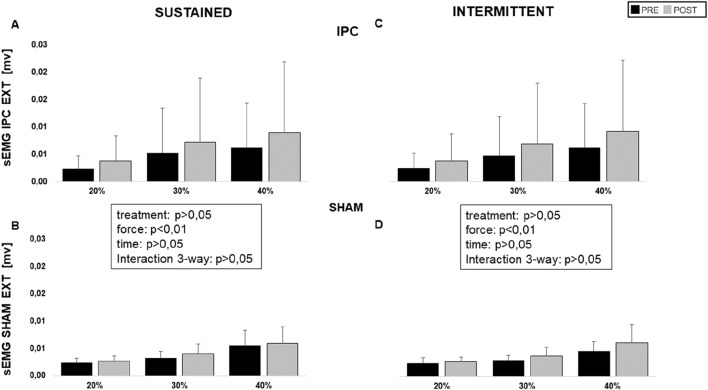
Average sEMG intensity reached by wrist flexor muscles in sustained **(A, B)** and intermittent **(C, D)** contractions at the different levels (20, 30, and 40% MVC) for the IPC **(A, C)** and SHAM **(B, D)** groups, before (black) and after (grey) treatment.

Original tracings from a representative subject are shown in Figure 2A. The required force level was easily maintained for the whole duration of the contraction by all subjects and exhibited no dependence on treatment session or time, with no three-way interaction between these factors and force level, and no simple two-way interactions: on average, 6.1 ± 1.9 kg, 8.9 ± 3.1 kg, and 11.9 ± 3.9 kg, at 20%, 30%, and 40% MVC, respectively. As for the reported perception of fatigue, it was only affected by the force level (p < 0.001) and not by treatment session or time, with no 3- nor 2-way interactions: on average VAS report was 2.3 ± 1.0, 2.8 ± 0.9, and 3.4 ± 0.9, at 20%, 30%, and 40% MVC, respectively. Average responses from all subjects are shown in Figure 3A, for the 40% MVC force level while the results of statistical analysis are reported in table n.1. In general, all parameters exhibited a dependence on force level but no effect of treatment session or time and no interactions, except EMG activity which exhibited a trend to increase with time in both extensor (p = 0.053, Figure 4 left) and flexor muscles (p = 0.091, Figure 5 left). In addition, the contraction-induced increase in deoxygenated haemoglobin was significantly larger in the IPC group compared to the SHAM, as also visible in Figure 3A with no effect of time and no time-treatment interaction.

### 5.3 Intermittent contractions

An example of original recordings from intermittent contractions is shown in Figure 2B. Also with intermittent contractions, the required force level was maintained for the whole duration of the task by all subjects and exhibited no dependence on treatment session or time, with no three-way interaction between these factor and force level, and no simple two-way interactions: on average 6.01 ± 1.9 kg, 8.4 ± 3.0 kg, and 11.5 ± 3.9 kg, at 20%, 30%, and 40% MVC. The perception of fatigue was only affected by the force level (p < 0.001) and not by treatment session or time, with neither 3- nor 2-way interactions: on average the VAS report was: 1.9 ± 0.8, 2.6 ± 0.9 and 3.1 ± 0.8, at 20%, 30% and 40% MVC, respectively. Figure 3B reports the average response curves from all subjects to intermittent contractions. Similar to what was reported for sustained contractions. Besides the significant main effect for force level on all analysed parameters, a significant effect for time is observed for EMG activity in extensor muscles (p = 0.01, Figure 4 right), just close to significance in flexors (p = 0.06, Figure 5 right), with no three-way or simple two-way interactions, while a significant time*force interaction is exhibited by EMG in flexor muscles (p < 0.05). As reported for the sustained contractions, also in this case the effect of treatment on HHb was close to statistical significance, with no effect of time and no time-treatment interaction (Table 1).

**TABLE 1:** Statistics. Main factors and interactions for the 3-way ANOVA over Force, EMG and NIRS variables (significant values as reported in bold): FORCE%: Force expressed as % of MVC; VAS: visual analog scale report of perceived effort; sEMG: average of the rectified surface electromyography signal; TOI_D_: tissue oxygenation index Delta; TOIs: tissue oxygenation index slope; HHb: deoxygenated hemoglobin; all referred to wrist flexors _(FLEX)_ and extensors _(EXT)_ muscles.

		time*force*treats	treats*force	treats*time	force*time	Treats	*force*	*time*
SUSTAINED	*Force%*	0.934	0.404	0.755	0.913	0.831	**0,001**	0.640
	*VAS*	0.542	0.974	0.630	0.667	0.930	**0,001**	0.820
	*sEMGFLEX*	0.291	0.417	0.154	0.470	0.509	**0,018**	0.096
	*sEMGEXT*	0.918	0.175	0.096	0.404	0.634	**0,001**	**0,053**
	*TOID-FLEX*	0.159	0.102	0.616	0.066	0.455	**0,01**	0.489
	*TOID-EXT*	0.723	0.313	0.735	0.709	0.305	**0,001**	0.728
	*TOIS-FLEX*	0.619	0.198	0.254	0.200	0.225	**0,034**	0.159
	*TOIS-EXT*	0.713	0.698	0.919	0.794	0.366	**0,01**	0.215
	*HHbD-FLEX*	0.856	0.228	0.748	0.556	0.197	**0,007**	0.973
	*HHbD-EXT*	0.365	0.091	0.337	0.070	**0,037**	**0,001**	0.137
INTERMITTENT	*Force%*	0.337	0.091	0.959	0.190	0.307	**0,001**	0.819
	*VAS*	0.163	0.393	0.599	0.349	0.998	**0,001**	0.128
	*sEMGFLEX*	0.546	0.382	0.316	**0,035**	0.485	**0,017**	**0,060**
	*sEMGEXT*	0.465	0.414	0.475	0.103	0.302	**0,001**	**0,01**
	*TOID-FLEX*	0.577	0.647	0.856	0.117	0.853	**0,001**	0.749
	*TOID-EXT*	0.508	0.307	0.647	0.142	0.673	**0,049**	0.896
	*TOIS-FLEX*	0.934	0.695	0.763	0.008	0.822	**0,009**	0.179
	*TOIS-EXT*	0.917	0.070	0.554	0.756	0.118	**0,024**	0.797
	*HHbD-FLEX*	0.677	0.470	0.873	0.289	**0,058**	**0,001**	0.585
	*HHbD-EXT*	0.409	0.572	0.354	0.371	**0,053**	**0,002**	0.327

## 6 Discussion

Our study explored the impact of Ischemic Pre-Conditioning (IPC) on local hemodynamic and sEMG responses during non-fatiguing voluntary contractions. The results evidenced no effect of IPC in electromyographic variables that could reveal the occurrence of an ergogenic effect, nor changes in systemic and local hemodynamic variables that could reveal an improvement of blood supply, in submaximal non-fatiguing contractions of either continuous or intermittent mode. EMG activity showed a trend to increase over time in both flexor and extensor muscles and in both SHAM and IPC groups. The changes in NIRS variables during contraction and hyperemia were not significantly affected by either the IPC or SHAM treatments.

### 6.1 Changes in EMG activity

Different data are found in the literature concerning changes in EMG activity induced by IPC; however, their interpretation requires careful consideration of the specific experimental conditions and protocols. An IPC-induced increase in EMG level was observed in a 60-s sprint exercise in recreational cyclists. It was associated with increased power output and increased lactate production, suggesting increased motor drive and increased recruitment of muscle fibers (Cruz et al., 2016). This possibility is supported by different possible mechanisms, including inhibition of group III and IV muscle afferents (Cruz et al., 2017) and placebo effects (Marocolo et al., 2023; Souza et al., 2021). An increase in EMG activity was also observed in a previous study in which, however, the power output was maintained constant and equal to a previously measured level (Cruz et al., 2015). While the effect was similarly interpreted and considered to explain the increase in endurance (time to exhaustion), we here stress the concept that increased EMG activity at constant force/power, on the contrary, may indicate weakened muscle contractility. In fact, in this case, the increase in motor drive may occur as the necessary compensation for the muscle-weakening to attain the same force level. The most common example of this phenomenon is probably the EMG increase that takes place with the progression of muscle fatigue, whereby a growing number of motor units is progressively recruited and their firing rate increased to maintain the predefined force level (Löscher et al., 1996). Another example is the increase in the firing rate of motor units that were observed in response to stress-induced muscle weakening during constant-torque isometric contractions (Roatta et al., 2008). We believe that the same reasoning explains the increase in EMG observed in the present study, following both IPC and SHAM treatments: the EMG increase would result from the adaptation of motor control (increased motor drive) to a progressive muscle weakening, possibly related to the long experimental protocol, as discussed below. This interpretation is supported by a recent investigation on IPC effects on electrically stimulated contractions of the adductor pollicis (Allois et al., 2024). It was observed that the force produced at different stimulation frequency (8–12 Hz) was slightly reduced after both IPC and SHAM treatments: a muscle weakening that appeared to be related to a shortening of the half-relaxation time of the single twitch, implying decreased twitch fusion in sub-tetanic contractions (Allois et al., 2024). We don’t know if the weakening is due to prolonged sitting (>1 h) reducing blood flow and/or changes in muscle temperature affecting twitch contraction (Bennett, 1984), or to other unknown factors. However, the temperature factor can probably be excluded, given that skin temperature was continuously monitored and changes with time were not detected. Conversely, a slight decrease in resting tissue oxygenation was observed, suggesting that a decrease in perfusion took place. Given the known dependence of muscle force on adequate blood supply (Fitzpatrick et al., 1996) and tissue oxygenation (Drouin et al., 2022) it is possible that the slight weakening of skeletal muscle was caused by a small reduction in resting blood flow and oxygenation. Notably, in both the present and the previous study (Allois et al., 2024), IPC did not alleviate the time-dependent weakening effect, on the contrary, it further slightly worsened it compared to SHAM. Similar results were observed in a recent study reporting weakening effects occurring in IPC, SHAM and control (no treatment) conditions, in terms of decreased maximal voluntary contraction and magnitude of the electrically-stimulated single twitch, in knee extensor muscles (Halley et al., 2018). All these data consistently negate any ergogenic action at the skeletal muscle level. In this respect, we further emphasize that, in the present context of submaximal non-fatiguing exercise, the observed EMG increase would not result from increased spinal excitability (by, e.g., humoral factors, alteration in the activity of group III and IV muscle afferents, etc.) but directly from adjustments of the voluntary motor command, required to match the muscle force with the visual target.

### 6.2 Hemodynamic monitoring

The present results indicate that contraction-related hemodynamic changes are not affected by the IPC or the SHAM treatment. Surprisingly, the HHb increase was on average higher in the IPC than the SHAM group (both in pre- and post-treatment exercise), while no difference was observed in the almost specular TOI decrease. We believe that this is a random effect possibly related to the location of optodes over the forearm, which is rich of superficial veins. In fact, contrary to what is generally believed, NIRS is quite sensitive to the presence of large vessels in the sample volume (Seddone et al., 2022). Moreover, it has been largely demonstrated that Beer-Lambert parameters such as HHb are particularly disturbed by skin blood flow (Messere and Roatta, 2013b) as well as by large superficial veins of the forearm (Messere and Roatta, 2015), while spatially-resolved parameters, like TOI, remain unaffected. Interestingly, other authors also reported a large difference in the HHb response to a 2-min MVC contraction, but, at difference with the present results, the HHb increase was higher in the IPC than in the SHAM group (+37%) (Halley et al., 2018). Although this was speculatively interpreted in terms of altered oxygen delivery, we believe it was as well due to random differences in sample volume, also considering that the HHb slope was unaffected by treatment and there were no differences in force performance and EMG activation. For these reasons, we consider of little importance that the magnitude of HHb response was different between IPC and SHAM groups in the pre-treatment condition while we emphasize that the response did not change after any of the treatments in terms of both magnitude of changes and slope of both TOI and HHb. HHb is a good marker of oxygen use (during ischemia) and fractional O2 extraction during steady exercise (Tuesta et al., 2022). It is also linked to muscle fatigue (Scano et al., 2020) and is less affected by changes in skin blood flow compared to O2Hb (Grassi and Quaresima, 2016). TOI is also considered for the same purpose (Tuesta et al., 2022). In general, the slope of HHb and TOI changes is proportional to the mismatch between perfusion and metabolism (Ferrari et al., 2011). Considering that IPC has been proposed to improve perfusion and decrease oxygen consumption (Allois et al., 2023; Paradis-Deschênes et al., 2016), lower slopes were expected in comparison to SHAM. In contrast with this assumption, some studies reported an increased slope after IPC (Kido et al., 2015; Tanaka et al., 2016) indicating increased oxygen consumption. However, the exercise load was generally increased after IPC thus accounting for the increased metabolic rate. In a previous study investigating the effect of IPC on fatiguing intermittent isometric contractions of the biceps muscle, we observed indeed a decrease in both the TOI and HHb response after IPC and not after SHAM, suggesting decreased oxygen consumption (Allois et al., 2023) in agreement with other studies (Orbegozo Cortés et al., 2015). These effects were not detected in the sustained non-fatiguing contractions of the present study. In addition, no difference was detected also in the intermittent contractions, which allow for periodic recovery of blood supply to the muscle, suggesting that IPC did not substantially improve vascular reactivity and muscle perfusion. This observation is in agreement with the absence of blood flow and hemodynamic changes reported for a rhythmic handgrip task to exhaustion (Barbosa et al., 2015); but positive results have also been reported, as reviewed in (O’Brien and Jacobs, 2022). These results support the concept that the benefits of IPC treatment may only appear in endurance tasks (Salvador et al., 2016), or close to exhaustion.

### 6.3 Ergogenic effects

Ergogenic effects are obviously of great interest in sport science for both endurance and strength specialties. Several studies report on the ergogenic effects of IPC. Most of them investigate complex sports settings (Crisafulli et al., 2011; Rodrigues et al., 2023; Salvador et al., 2016) in which the performance improvement may result from multiple sources, including the cardiovascular and respiratory systems, possibly affected by remote IPC (Hausenloy and Yellon, 2008). In laboratory studies, investigating muscle performance under more controlled settings, e.g., isometric contractions (Allois et al., 2023; Bailey et al., 2012), the force improvements may still arise from local (e.g., muscle metabolism, tissue oxygenation, contractility) and central factors (e.g., arterial blood pressure, motor drive, spinal excitability, placebo effects). We here adopted low-level, non-fatiguing, voluntary contractions to possibly detect local changes in the relevant muscles, excluding the contribution of central effects by requiring to maintain the same submaximal force level pre- and post-treatment. The results failed to evidence any local ergogenic effect of IPC: no increase in contractility, which would have produced a reduction in EMG levels; no increase in blood perfusion, which would have produced an increase in resting tissue oxygenation; no decrease in oxygen consumption, which would have reduced the slopes of TOI and HHb. The results are in line with our previous study on electrically-evoked contractions (Allois et al., 2024) and agree with others that failed to evidence improvement in muscle force or performance, although most of them did not specifically focus on peripheral effects (Halley et al., 2018; Souza et al., 2021; Valenzuela et al., 2021).

In conclusion, the present study demonstrates the absence of peripheral ergogenic effects of IPC in the treated muscles, in submaximal non-fatiguing contractions. Of course, this does not negate the possibility that ergogenic effects can be achieved via central mechanisms that may potentiate the motor command to skeletal muscles (O’Brien and Jacobs, 2021). These include the placebo effect, and other mechanisms increasing the excitability of motor circuits at the spinal level, such as reduced afferent feedback by group III and IV muscle afferents (Crisafulli et al., 2011; O’Brien and Jacobs, 2022) although it has been shown that this may also limit cardiovascular support to physical activity (Angius et al., 2022). The occurrence of these enhancing effects on the motor drive can be unmasked only during maximal efforts. In addition, the present investigation does not exclude that additional mechanisms improving perfusion and metabolism efficiency may become apparent and explain IPC benefits in long-lasting fatiguing contraction and endurance exercise.

### 6.4 Limitations

Whether the SHAM treatment (20 mmHg compression) may effectively act as a placebo is often questioned since the difference from the IPC treatment (250 mmHg) is clearly perceived by the subjects. Although the occurrence of a placebo effect cannot be completely excluded, we believe it played no role in the results, considering that: 1) it was said to the subjects that the ischemic and slightly painful IPC treatment could either improve or worsen the muscle function and 2) the exercise task did not include maximal strength or endurance efforts but non fatiguing and short lasting exercises that are, by design, always performed in the same way (matching the visual and audio feedbacks), irrespective of real ergogenic or placebo effects.

## 7 Conclusion

This study investigated peripheral IPC effects on continuous and intermittent voluntary contractions of non-fatigued muscles. No evidence of ergogenic effect was detected based on the analysis of EMG and hemodynamic signals, confirming previous observations on electrically stimulated contractions, The results suggest that ergogenic benefits of IPC are limited to potentiation of the motor drive during maximal efforts or strenuous endurance exercise. The ergogenic effects of IPC have been observed in various sports, but despite extensive research, the actual benefits and mechanisms remain unclear, with the placebo effect being identified as a significant factor. This study examined sub-maximal, non-fatiguing contractions to explore potential changes in muscle contractility and blood flow control. No changes in muscle contractility or improvements in vascular reactivity or tissue oxygenation were found. Therefore, any ergogenic effects in this context may only result from increased motor drive, highlighting the need to consider the placebo effect when studying IPC’s ergogenic impact. Analyzing IPC effects by differentiating between local and central mechanisms and fatigue/non-fatigue muscle conditions may improve our understanding of IPC’s ergogenic effect and its best application in sport and exercise.

## Data Availability

The raw data supporting the conclusions of this article will be made available by the authors, without undue reservation.

## Abbreviations

ABP, Arterial Blood Pressure; ANOVA, Analysis Of Variance; FORCE%, Percentage levels Force in sustained and intermittent contractions; HHb_FLEX_, Hemoglobin + Myoglobin Deoxygenated in flexors muscle; HHb_EXT_, Hemoglobin + Myoglobin Deoxygenated in extensors muscle; IPC, Ischemic Pre-Conditioning; MVC, Maximum Voluntary Contraction; NIRS, Near-Infrared Spectroscopy; sEMG_FLEX_, superficial electromyography signals of the flexors muscle; sEMG_EXT_, superficial electromyography signals of the extensors muscle; TEMP, Temperature; TOI, Tissue Oxygenation Index; TOI_B-FLEX_, Tissue Oxygenation Index Baseline in the flexors muscle; TOI_B-EXT_, Tissue Oxygenation Index Baseline in the extensors muscle; TOI_D-FLEX_, Tissue Oxygenation Index Delta in the flexors muscle; TOI_D-EXT_, Tissue Oxygenation Index Delta in the extensor muscle; TOIs_-FLEX_, Tissue Oxygenation Index Slope in the flexors muscle; TOIs_-EXT_, Tissue Oxygenation Index Slope in the flexors muscle; VAS, Visual Analogic Scale.
